# A Few Notes Concerning the Progress of Dental Surgery

**Published:** 1858-06

**Authors:** Geo. H. Perine

**Affiliations:** New York


DENTAL REGISTER OF THE WEST.

VOL. XI.—JUNE, 1858.—No. 4.

### Original Essays and Communications.

### A FEW NOTES CONCERNING THE PROGRESS OF DENTAL SURGERY.

BY GEO. H. PERINE, NEW YORK.

The origin of this profession is lost in the obscurity of the past. We have, however, data proving its existence as a separate branch of study at a very early period; and many curious facts which we have been enabled to collect concerning its progress, may not prove uninteresting to our readers. Among the ancient Egyptians, drawing teeth was confined to a special class. That they not only drew teeth, but also filled them, is proved to us by the existence of teeth in good preservation in the skulls of mummies, bearing evidence of having been filled with various aromatic preparations, and even with gold. These teeth also bore marks of the file upon their surface, thus leading us to believe that the Egyptians had arrived at greater perfection in instrument manufacture than is generally supposed. Herodotus also speaks of Dentists as existing among the Egyptians. Esculapius, the father of all medicine, is said to have first practiced as a dentist. At that time, having no means for supplying teeth when lost, people were naturally unwilling to have them drawn, until necessity compelled them. Erasistratus mentions an Odontague, or instrument for extracting teeth, which hung suspended in the temple of Delphos. It was made of lead, thus signifying that no teeth should be drawn, which would require a stronger metal. Hippocrates also recommends that the rule be followed in all cases, advising cauterization when the tooth is much decayed. Heraclides, of Tarentum, and others, speak of deaths having occurred from the careless extraction of teeth.

Various compositions existed in those days for the preservation of the teeth. Hippocrates mentions a remedy for bad breath and discolored teeth; and Memacus a preventive of hollow teeth. Celsus speaks of filling the teeth with alum enclosed in cotton, which eased the pain, while it preserved the tooth. Dentifrice, or tooth power, was then in vogue, as now; Damocrates, and Octavia, sister of Augustus, each are known as inventors. Many strange remedies were then popular among those unfortunate enough to have decayed or hollow teeth. Some used vapor baths, others cautery, while many wore amulets to prevent pain. One distinguished disciple of the Dental Art, gives the following recipe as a sure and unfailing safeguard against the toothache for one year: "To preserve the teeth from pain or disease for a year, let the patient, on the appearance of the first swallow of the season, proceed to the bank of a clear river, and thus address the potent bird, after having first filled his mouth with the clear water of the stream, and washed his teeth with the index finger of each hand: 'Hirudo, tibi dico, quo modo hoc in nostro iterum non erit, sic mihi dentes non doleant toto anno!'" Whether this preventive achieved a success or not, is not recorded. Injections into the nose and ear were also customary remedies in the pharmacopœa of the Ancients. The Arabs followed the Greeks in their medical practice. For a long period the practice of Dentistry was confined wholly to the bath-keeper; but the many accidents resulting from their unskillful treatment caused to be created a separate profession of Dental Surgery, first existing under Abu'l Kasem, who was also the first to replace drawn teeth by others, sometimes natural, at others manufactured from ox bones.

Being cowardly by nature, and especially fearful of bodily pain, the Arabs gladly availed themselves of every remedy which promised relief without pain. Aaron, a physician of Alexandria, proposed for this purpose, colocynth, a bitter apple. Hhonam Ebn Izhak, and Rhazes also proposed various similar preparations. Abu'l Kasem was the first who remarked the collection of tartar upon the teeth.

Although we have thus furnished sufficient evidence to prove that disease existed in the teeth of the Ancients, as well as with us, yet we can not consider it as prevalent as in more modern times. What appears to have been then the exception, is now the rule. In the excavations at Pompeii, in what appeared to be a prison attached to a barrack, the skeletons of some soldiers were found in chains; and in their skulls were found teeth perfectly preserved, though two thousand years buried in the earth. Many such facts have been discovered, which, taken with our knowledge that the Ancients had no sugar, lead us to believe, and justly, that their teeth were much better preserved than are those of our time. The origin of the invention of artificial teeth is thus stated:—Among the ancient Egyptians, it was customary to punish offenders, in certain slight cases, by extracting a tooth. As they were thus branded, and shunned by every one, they hit upon the expedient of forming artificial teeth of various substances, and filling with them the disgraceful cavities.

The writers of the Middle Ages do not refer to dental operations, until Bruno, of Longobucco, who expresses great faith in the powers of red-hot iron, with which he cauterized cancers, diseased gums, and teeth. He did not approve of extraction, but recommended the use of certain drugs, and natural substances, which finally caused the tooth to fall out. Among these, the most powerful was the fat of the Rennet, a species of frog. This was so effective, that it is related of a cow which accidentally took one into her mouth while pasturing, that she lost all her teeth in consequence.

Guy de Chaulioc, a medical writer of the same time, complains of the custom of entrusting the practice of Dentistry to the hands of illiterate and careless barbers: thinking it as important as any other branch of the science. At this period, we learn that narcotics were used during the extraction of teeth, to assuage pain; thus shadowing forth the more modern potency of chloroform and ether in similar operations. Guy de Chaulioc, it is said, filled the cavities of decayed teeth with resin and other aromatic substances, to prevent their becoming clogged with food.

A prevalent theory explanatory of the cause of dental disease at this time, was the existence of worms. Many dentists attest the truth of this theory. Worms were said to have been taken from the teeth measuring one and a half inches in length. Many strange plans were proposed for the wholesale murder of these insatiate monsters, which generally, however, if we may believe the chroniclers, resulted in the slaughter of those patients unfortunate enough to be the subjects of treatment by the unskillful and superstitious surgeons of the age. John Arcularius, at this time, filled teeth with gold; and shortly after, appeared the first work devoted to Dentistry, by Gautier Henri Ryff; but we have been unable to discover any trace even of its title. Later, Strobelberger advised the smoking of tobacco, then newly discovered. Dupont counselled the extraction of diseased teeth, and their immediate replacement; they soon fastened themselves anew. Denis Pomeret confirms this, and himself replaced a sound tooth drawn by mistake. According to his own account, he was completely successful.

John Scultet, at this period, invented many new instruments for dental surgery. Gauthier Harris suggested lancing, in cases of difficult dentition; but it was not extensively practiced.

This brings us to a more modern period, with the dental history of which our readers are, doubtless, acquainted. The rapid strides the profession has taken in the last century have placed it in the front rank of science. In this profession Americans stand preëminent, and they are found established, in extensive and lucrative practice, in all the courts of Europe. In the manufacture of teeth the wealth of the earth, vegetable and mineral, has been pressed into service; and the result has been in the highest degree successful. For durability, power to perform their allotted work, and delicacy of finish, the efforts of dental art may compare favorably with those of any other.

We will conclude this article with some curious facts which we have collected, not necessarily forming a portion of dental history, but interesting in themselves, as proving that even in a subject so utilitarian as Dentistry, there may be found "facts stranger than fiction."

From an intelligent and educated young native of China, we learn several particulars concerning the practice of dentistry in that country, which, like everything else relating to the inner life of the Celestials, is tinged with the ludicrous. The Chinese take no care of the teeth. Among them, toothbrushes are an article of luxury seldom indulged in; yet, strange to say, there are none who suffer less from toothache. As there is little necessity for dentists, there are few who learn the profession; and the knowledge of those who do, extends no farther than extracting teeth. Unlike their brethren of this country, Chinese Dentists are generally so poor that they can not afford to keep a house wherein to operate; but in place of it, have a little stand which they trundle round the streets like our itinerant beer-carts on Independence-day. Around the outside of the cart are strung rows of teeth, showing the amount of business which has been done by the dentist. At Shanghae, an open park around the great Temple is devoted to the amusements and the street business of the city. There collect the jugglers with their wonderful feats; there are the tea-houses, where the Celestial swain leads his "fayre ladye" to revel in the "cup that cheers but not inebriates." And here, too, the traveling dentists "most do congregate;" and the air is filled with shouts of laughter from those seeking the amusements of the place in one moment, and the next, perhaps, with the screams of the unfortunate, under the hands of the tooth-puller.

Next we note a few "dental superstitions" and miscellaneous facts:

"Teeth set wide apart are said to be a sign that their possessor will be lucky, and will travel much."

"When the upper incisors are large, it is a sign of future wealth."

*Cure for the toothache.*—"Watch a mole-trap, and when it springs, take the mole therefrom while living, cut off his paws, and wear them as amulets,—if the aching proceed from the right side of the jaw, wear the dexter paw, and vice versa. A mole's paw mounted in silver has been seen among other relics, in the possession of a family in London." This is an ancient English superstition.

In "Blagrave's Mathematical Jewel," London, 1585, is stated among other things concerning the author, that his nephew, Sir John Blagrave, "caused his teeth to be all drawne out, and after had a sett of ivory teeth in againe."

In Ben Jonson's "Silent Woman," Act IV, Sc. I, Otter, speaking of his wife, says, "A most vile face! and yet she spends me forty pounds a year in mercury and hog's bones; all her teeth were made in the Black-Friars!"

In London there existed a class of men, who, for a certain price, permitted their teeth to be drawn, and inserted in the jaws of those who had lost their own by disease or otherwise.

Bell, Saunders, and others, state that the age of children, from seven years until thirteen, can be accurately ascertained by examining their teeth.

In connection with cases of longevity, many strange facts are related concerning dentition at an advanced period of life. These facts would appear to indicate the possession of the "Elixir Vitæ" of the Ancients; and we can hardly blame the subjects of this singular rejuveniscence for imagining that their long-lost youth was returned.

In a common-place book. written by one Thomas Rawlins, of Pophills, England, between the years 1724 and 1734, occur the following entries:

"There lived in Mill street in Belfast, in Ireland, 1731, one Jane Hooks, of one hundred and twenty years of age, who has her memory and appetite as well as when she was but twenty years old, and has got a new sett of teeth w$^{ch}$ has drove out all y$^{e}$ old stumps."

"Rob't Lyon of y$^{e}$ city of Glasgow, aged one hundred and nine years, who was in the service of Charles I., and who has got a new set of teeth and recovered his sight in a wonderful manner."

"Mrs. Page at y$^{e}$ Royal Oak in Barnaby street, Southwark, aged ninety years and upward, has lately bred six great teeth in y$^{e}$ upper jaw in June, 1732, which is an extraordinary and preternatural instance, had not a tooth in her head these twenty years."

"Margaret White, of Kirkaldy in Scotland, aged eighty-seven, who had been toothless for many years, has just got eight new and fresh teeth. April, 1732." CH. HOPPER.

"Hon. Edward Progers, of London, died Dec. 31st, 1712, aged ninety-six, of the anguish of cutting teeth; he having cut four new teeth, and had several ready to cut, which so inflamed his gums that he died thereof."

Three hundred years ago, Martin Luther complained of the toothache; and a German ambassador at the Court of Queen Elizabeth mentions the imperfection of the teeth of the English people, attributing their weakness and disease to the prevalent habit of eating sugar. Roger Williams was astonished to find the Narragansett Indians complaining seriously of toothache. George Washington wore artificial teeth—Walter Scott complains of toothache, while Napoleon at St. Helena suffered much from "the aching fang." And, finally, we can not better close our hotch-potch of dental notes, than by quoting Robert Burns' pathetic "Address to the Toothache."

> "When fevers burn, or ague freezes,
> Rheumatics knaw, or colic squeezes,
> Our neighbors' sympathy may ease us,
> W' pitying moan;
> But thee, thou hell o' a' diseases,
> Ay mocks our groan!
> Where'er that place be priests ca' hell,
> Whence a' the tones o' mis'ry yell,
> And ranked plagues their numbers tell,
> In dreadfu' raw,
> Thou, Toothache, surely bear'st the bell
> Amang them a'!"

